# Focused View CT Urography: Towards a Randomized Trial Investigating the Relevance of Incidental Findings in Patients with Hematuria

**DOI:** 10.3390/diseases13080242

**Published:** 2025-08-01

**Authors:** Tim E. Sluijter, Christian Roest, Derya Yakar, Thomas C. Kwee

**Affiliations:** Medical Imaging Center, Department of Radiology, University of Groningen, University Medical Center Groningen, 9700 RB Groningen, The Netherlands; t.e.sluijter@umcg.nl (T.E.S.); c.roest@me.nl (C.R.); d.yakar@umcg.nl (D.Y.)

**Keywords:** hematuria, urography, incidental findings, artificial intelligence

## Abstract

**Background:** Computed tomography urography (CTU) is routinely used to evaluate the upper urinary tract in patients with hematuria. CTU may detect incidental findings outside the urinary tract, but it remains unclear if this adds value. This study aimed to develop a deep learning algorithm that automatically segments and selectively visualizes the urinary tract on CTU. **Methods**: The urinary tract (kidneys, ureters, and urinary bladder) was manually segmented on 2 mm dual-phase CTU slices of 111 subjects. With this dataset, a deep learning-based AI was trained to automatically segment and selectively visualize the urinary tract on CTU scans (including accompanying unenhanced CT scans), which we dub “focused view CTU”. Focused view CTU was technically optimized and tested in 39 subjects with hematuria. **Results**: The technically optimized focused view CTU algorithm provided complete visualization of 97.4% of kidneys, 80.8% of ureters, and 94.9% of urinary bladders. All urinary tract organs were completely visualized in 66.6% of cases. In these cases (excluding 33.3% of cases with incomplete visualization), focused view CTU intrinsically achieved a sensitivity, specificity, positive predictive value, and negative predictive value of 100.0%, 92.3%, 92.9%, and 100.0% for lesions in the urinary tract compared to unmodified CT, although interrater agreement was moderate (κ = 0.528). All incidental findings were successfully hidden by focused view CTU. **Conclusions**: Focused view CTU provides adequate urinary tract segmentation in most cases, but further research is needed to optimize the technique (segmentation does not succeed in about one-third of cases). It offers selective urinary tract visualization, potentially aiding in assessing relevance and cost-effectiveness of detecting incidental findings in hematuria patients through a prospective randomized trial.

## 1. Introduction

Hematuria accounts for over 20% of urology visits, and microhematuria prevalence ranges from 2.4% to 31.1% in healthy individuals, depending on the population studied [1,2]. Bladder carcinoma is present in 15.0–20.0% of cases with visible hematuria (VH) and in 1.6–3.1% of subjects with non-visible hematuria (NVH). Carcinoma of the upper urinary tract (ureters and kidneys) is present in 1.5–3.3% of cases with VH and in 0.3–0.9% of subjects with NVH [3].

The diagnostic work-up of hematuria often involves cystoscopy for the evaluation of the bladder and imaging for the assessment of the ureters and kidneys, depending on the patient’s risk profile (VH and age > 50 years being the most important parameters), as well as the patient’s and provider’s preferences, institutional protocols, and available resources. Imaging of the upper urinary tract is often performed with computed tomography urography (CTU), given its high spatial resolution and widespread availability, while some institutions also use magnetic resonance imaging for this purpose due to its lack of radiation exposure and improved soft tissue contrast [4,5].

CTU may be useful to detect upper urinary tract cancer, but it also visualizes all other organs between the xiphoid process and pubic symphysis. Although the latter may be useful for metastatic disease assessment in urinary tract cancer, the majority of patients with hematuria have no urinary tract cancer, and in these cases, there is no clear medical reason to visualize the entire abdomen outside the urinary tract. Clinically significant incidental findings (i.e., newly detected asymptomatic findings which are not clearly benign and may require treatment to prevent morbidity or mortality [6]) are detected on 7.2–15.0% of CTU examinations performed for the evaluation of hematuria [4,5,7]. These incidental findings can lead to additional diagnostic procedures, increased healthcare costs, patient anxiety, and potential overtreatment [5,8,9]. Whether or not the detection of incidental findings in this setting is useful in terms of health gains and cost savings remains unknown.

With new deep learning-based artificial intelligence (AI) techniques, it has become possible to automatically segment organs of interest with increasing accuracy and efficiency [10,11]. Deep learning-based segmentation algorithms may be used to selectively display the urinary tract on CTU examinations while eliminating all other organs that may contain incidental findings, a technique we dub “focused view CTU”. This approach could minimize diagnostic distractions and reduce follow-up testing prompted by incidental findings. It would also allow a prospective clinical trial to be conducted, in which patients with hematuria are randomized to undergo either conventional CTU or focused view CTU, with the goal of assessing the clinical utility, downstream consequences, and cost-effectiveness of incidental findings detection. However, an algorithm specifically designed and validated for focused view CTU has not yet been developed, representing a current gap in the field. We hypothesize that deep learning-based focused view CTU will enhance urinary tract visualization while reducing incidental findings and related unnecessary follow-up procedures.

The purpose of this study was to develop a new deep learning-based algorithm that automatically segments and selectively visualizes the urinary tract on CTU.

## 2. Materials and Methods

### 2.1. Study Design and Subjects

This retrospective study was approved by the institutional review board of the University Medical Center Groningen and the requirement for informed consent was waived because of the use of anonymized data.

**Figure 1:**
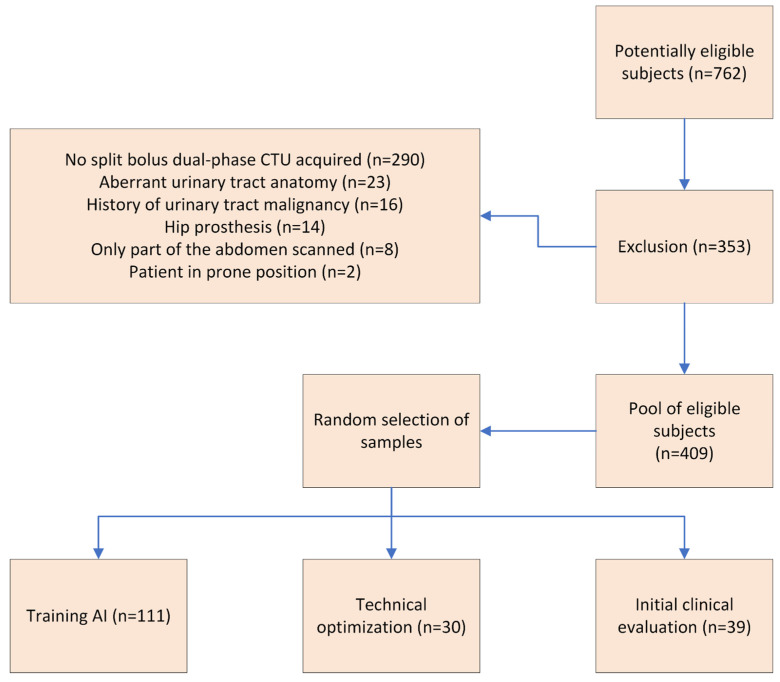
Patient selection flowchart. The following pre-defined inclusion criteria were used: all CTU scans performed because of hematuria at our tertiary care center between 1 January 2018 and 31 December 2021. The following pre-defined exclusion criteria were used: CTU not performed using the split-bolus contrast technique combining nephrographic and excretory phases (i.e., dual-phase CTU); abnormal urinary tract anatomy (e.g., post-cystectomy, kidney transplantation, single kidney, or extensive ascites compressing the bladder); history of urinary tract cancer; presence of a hip prosthesis (which introduces artifacts near the bladder); incomplete abdominal coverage (i.e., not extending from the xiphoid process to the pubic symphysis); non-supine patient positioning during CTU; and presence of a ureteral stent.

The following pre-defined inclusion criteria were used: all CTU scans performed because of hematuria at our tertiary care center between 1 January 2018 and 31 December 2021. The following pre-defined exclusion criteria were used: CTU not performed using the split-bolus contrast technique combining nephrographic and excretory phases (i.e., dual-phase CTU); abnormal urinary tract anatomy (e.g., post-cystectomy, kidney transplantation, single kidney, or extensive ascites compressing the bladder); history of urinary tract cancer; presence of a hip prosthesis (which introduces artifacts near the bladder); incomplete abdominal coverage (i.e., not extending from the xiphoid process to the pubic symphysis); non-supine patient positioning during CTU; and presence of a ureteral stent. These cases were excluded as they could significantly impact image quality and segmentation accuracy. This provided a pool of 409 subjects, from which random samples were drawn for the purpose of this study (Figure 1), ensuring representative and unbiased selection.

### 2.2. CTU Parameters

CT scanning was performed using four different CT systems (SOMATOM Force, SOMATOM Flash, SOMATOM Edge, or SOMATOM Definition AS 64; Siemens Healthineers, Erlangen, Germany). Both unenhanced CT scans and dual-phase CTU scans were acquired from the xiphoid process to the pubic symphysis, ensuring comprehensive imaging coverage of the entire urinary tract anatomy. Immediately before the CTU, 20 mg of Furosemide was administered intravenously. Subsequently, 85 mL of Iomeron 350 mixed with 20 mL of saline was administered intravenously at an injection rate of 1 mL/s. After 12 min, another 50 mL of Iomeron 350 mixed with 20 mL of saline was administered intravenously, followed by the CTU scan. All scanners used the following settings: tube voltage of 100 kV for unenhanced CT, 120 kV for dual-phase CTU; gantry rotation time of 0.5 s; collimation of 0.6 mm. Automated exposure control was switched on during acquisitions on the SOMATOM Flash (CARE Dose 4D; Siemens Healthineers). Iterative reconstruction was applied (ADMIRE, Siemens Healthineers) and set to 5 on unenhanced CT and set to 4 on dual-phase CTU. A reconstructed slice thickness of 2 mm was used for further postprocessing and analysis. All scans were acquired with the patient in supine position.

### 2.3. Manual Segmentation

**Figure 2:**
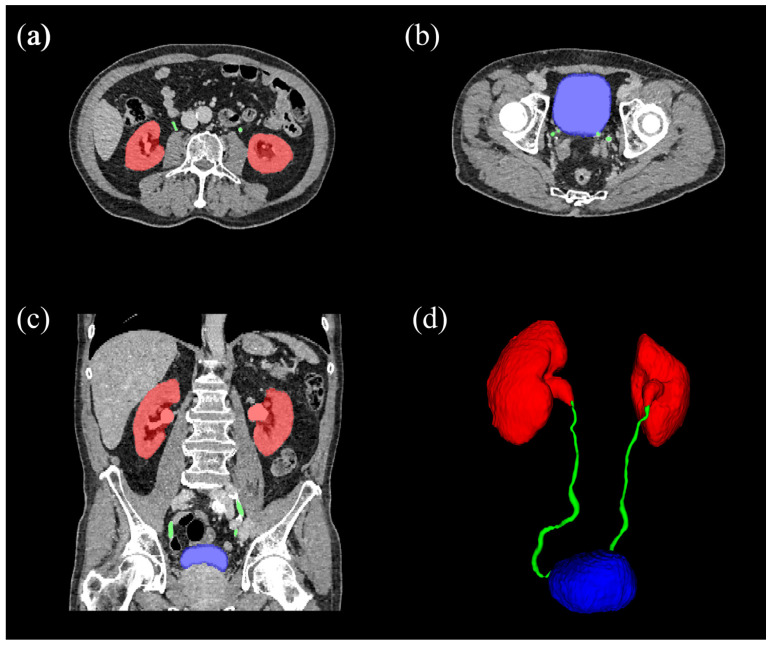
Example of a manual segmentation overlayed on CTU, showing voxels segmented as kidneys (red), ureters (green), and bladder (blue). Subfigures show axial slices of the kidneys (**a**) and bladder (**b**), and a coronal slice containing all three segmented structures (**c**). A 3-dimensional rendering of the segmentation is shown in (**d**).

A total of 111 CTU scans were randomly selected from the aforementioned pool of subjects for manual segmentation of the urinary tract. A research fellow (T.E.S., a medical doctor-researcher without specialty training), who was supervised by a radiologist (T.C.K.), manually segmented the kidneys, ureters, and urinary bladder on 2 mm dual-phase CTU slices, using ITK-SNAP version 4.0.1 available from www.itksnap.org (accessed on 1 July 2022) [12]. An example of a manual segmentation is shown in Figure 2.

### 2.4. Deep Learning Segmentation

The full manually annotated dataset of 111 scans and segmentations was used to train a deep learning-based AI algorithm capable of accurately and automatically segmenting the kidneys, ureters, and bladder on CTU scans. Cross-validation was used to train five separate U-Net models. The models were trained by optimizing the categorical Dice loss, with an equal weighting for each class during training. The U-Net had an input size of 192 × 160 × 128 at a resampled voxel spacing of 1 × 1 × 2 mm^3^, resulting in a field of view of 192 × 160 × 256 mm^3^, enabling a full view of the upper urinary tract. To enable full-scale segmentation without downsampling or resizing the scans, the model was trained on randomly sampled scan crops, and a sliding window approach was adopted for inference. The RMSProp optimizer was used with a learning rate of 10^−4^. The validation performance was tracked during training by calculating the loss on the validation portion of each fold. After 250 training epochs were completed, the model corresponding to the epoch with the best validation loss was selected as the best model. The final models for each fold were combined in an averaging ensemble to improve consistency in the predictions. The ensemble was subsequently applied to the testing data to generate segmentations for each testing scan. The deep learning pipeline was implemented using TensorFlow 2.7.1 and trained on a 32GB Nvidia V100 GPU.

### 2.5. Technical Optimization

**Figure 3:**
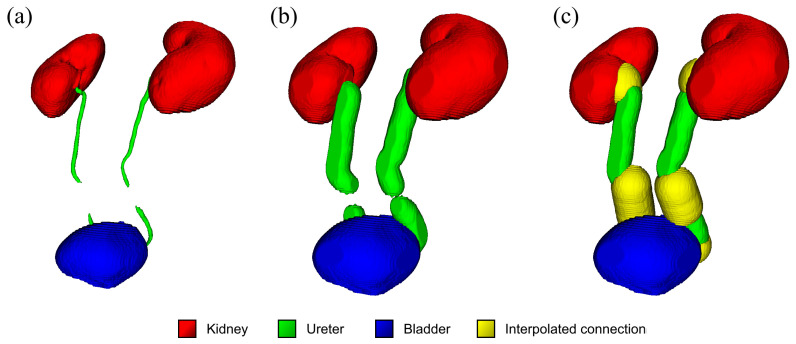
Visual representation of method 1 (**a**), method 2 (**b**), and method 3 (**c**) for generating focused view CTU (method 1: the baseline method, in which the unmodified AI-generated segmentation is used to mask the voxels outside of the segmented regions; method 2: the expanded boundary method in which a binary dilation with a spherical structuring element is used to expand the visualized area with a 10 mm boundary around the segmented; and method 3: the expanded boundary and extrapolation method, which expands on method 2 by bridging interrupted segmentations of the ureters by ensuring that the segmentation contained a connection between both kidneys and the bladder).

Three different methods for generating focused view CTU were developed: (1) the baseline method, in which the unmodified AI-generated segmentation was used (method 1) (Figure 3a); (2) the expanded boundary method, in which a binary dilation with a spherical structuring element was used to expand the visualized area with a 10 mm boundary around the segmented regions (method 2) (Figure 3b); and (3) the expanded boundary and extrapolation method (method 3), which expands on method 2 by bridging interrupted segmentations of the ureters by ensuring that the segmentation contained a connection between both kidneys and the bladder. The latter approach was implemented by adding a straight path from each disconnected ureter component to the nearest other component until all components were connected. Connections made in this way were expanded with a 20 mm radius, offering an expanded area of visibility where the ureter could not be segmented by the AI (Figure 3c). Focused view versions of the CTU and unenhanced CT scans were created by masking all voxels outside of the segmented volume of interest. Rigid registration was applied prior to the application of the mask, to minimize potential misalignments between CTU and unenhanced CT (e.g., due to movement of the patient). Registration was performed by optimizing, through gradient descent, an affine transformation maximizing the cross-correlation between each pair of CTU and unenhanced CT. This process was performed automatically, with no manual corrections applied. The set of focused view CTU and focused unenhanced CT was simply referred to as “focused view CTU” in the remainder of the manuscript. The generation of focused view CTU masks was implemented in Python 3.9.6, using NumPy 1.21.3 and scipy 1.7.1. Image registration was implemented using SimpleITK 2.2.0.

Thirty new cases were randomly selected from our pool of subjects. The three different focused view CTU methods were applied to these cases. The visibility of each kidney; proximal ureter (the upper half of the ureter extending from the renal pelvis to the midpoint between the renal pelvis and its intersection with the external iliac artery); mid ureter (the lower half of this segment; extending from the midpoint to the level of the external iliac artery); distal ureter (ureteral tract between the intersection with the external iliac artery and the urinary bladder); and urinary bladder was assessed by a research fellow (<BLINDED>) on each of the three different CTU methods; using a 4-point scale (1 ≤ 75% visualization; 2 = 75–95% visualization; 3 = 96–99% visualization; and 4 = 100% visualization). Evaluations were performed in separate sessions, with a wash-out period of two weeks. All interpretations of the scans were similar. Visualization scores were compared between the three different CTU methods using the Friedman test with subsequent pairwise comparisons, for which the significance values were adjusted by the Bonferroni correction for multiple tests. The best focused view CTU method (i.e., the one with the highest visualization scores) was used for the initial clinical evaluation. *p*-values < 0.05 were considered statistically significant. Statistical analyses were performed using IBM SPSS version 28.0 (IBM Corp., Armonk, NY, USA).

### 2.6. Initial Clinical Evaluation

In total, 39 new cases (19 with a likely cause for hematuria and 20 without a visible cause for hematuria, and in total 17 of those with an incidental finding outside the urinary tract, according to unmodified unenhanced CT and dual-phase CTU) were randomly selected from our pool of subjects. The best of the three focused view CTU methods was applied to these cases.

A radiologist (T.C.K., with more than 5 years of post-residency experience interpreting CTU scans) assessed the focused view CTU scans, without knowledge of the original findings and other clinical and follow-up data. Visibility of each kidney, ureter, and urinary bladder was assessed using a 3-point scale (1 = substantial parts not visible, risk of missing pathology, 2 = miniscule parts not visible, negligible risk of missing pathology, 3 = completely visible). The presence of lesions in each kidney, ureter, and urinary bladder was assessed using a 5-point scale (1 = very unlikely, 2 = unlikely, 3 = unclear, 4 = likely, and 5 = very likely, with scores of 4 and 5 regarded as positive). The presence of incidental findings was also noted. Results were descriptively analyzed (image quality and incidental findings). The diagnostic performance (sensitivity, specificity, positive predictive value (PPV), and negative predictive value (NPV)) of focused view CTU for the presence of lesions in the urinary tract, compared to the unmodified CTU, was determined. The original unmodified CTU report was used as reference standard. A second radiologist (D.Y., with more than 5 years of post-residency experience interpreting CTU scans) independently evaluated all focused view CTU scans to determine interobserver agreement, calculated with κ statistics. All other reported results belong to those of the first observer. Please note that the decision to use the results of observer 1 as the primary data to be reported was made beforehand, without knowledge of how the two observers would perform. All interpretations of the scans were similar.

## 3. Results

### 3.1. Technical Optimization

**Figure 4:**
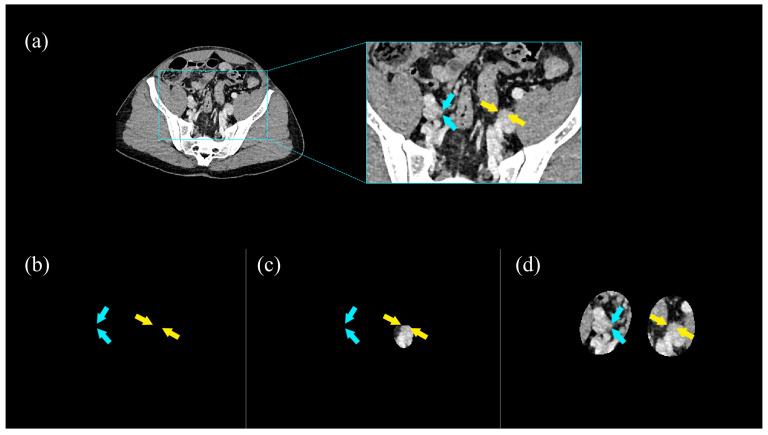
Example of parts of the right ureter (blue arrows) and left ureter (yellow arrows) shown on unmodified CTU (**a**), and on focused view CTU method 1 (**b**), method 2 (**c**), and method 3 (**d**), with method 3 as the focused view CTU approach that provides the best visualization of the ureters (method 1: the baseline method, in which the unmodified AI-generated segmentation is used to mask the voxels outside of the segmented regions; method 2: the expanded boundary method in which a binary dilation with a spherical structuring element is used to expand the visualized area with a 10 mm boundary around the segmented; and method 3: the expanded boundary and extrapolation method, which expands on method 2 by bridging interrupted segmentations of the ureters by ensuring that the segmentation contained a connection between both kidneys and the bladder).

**Figure 5:**
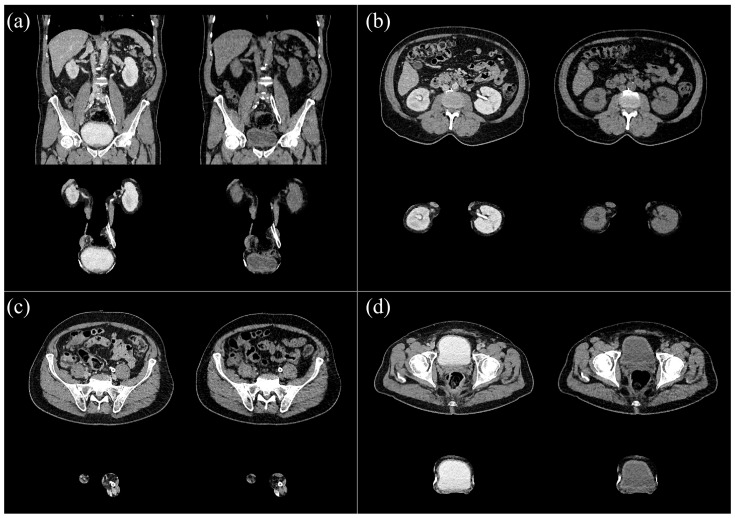
Example of the performance of focused view CTU method 3 (expanded boundary and extrapolation method) on coronal slices (**a**) and several axial slices (**b**–**d**), with contrast-enhanced and unenhanced CT images shown in each panel.

Overall, method 3 emerged as the best focused view technique, significantly outperforming the baseline and expanded boundary method on both CTU and unenhanced CT scans on most comparisons (Table 1, Figure 4 and Figure 5, and Supplementary Video Files S1 and S2).

### 3.2. Initial Clinical Evaluation

**Figure 6:**
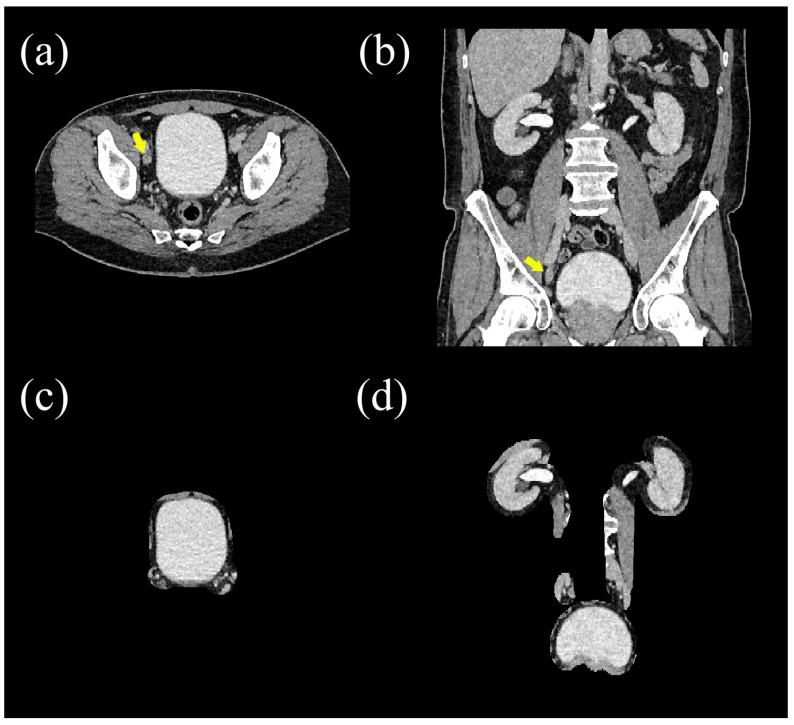
Example of an incidental finding (enlarged right para-iliac lymph node, indicated with an arrow on unmodified axial CT (**a**) and coronal CT (**b**)) which was successfully concealed on focused view CTU (**c**,**d**), allowing for prospective follow-up in a trial investigating the relevance of incidentalomas.

On focused view CTU (created using the method 3), almost all kidneys and bladders, and about 80% of ureters, were completely visualized. Substantial parts were not visible in approximately 15% of ureters (Table 2). On a subject level, all urinary tract organs were completely visualized in 26 out of 39 cases (66.6%). In the 26 cases with complete visualization of the urinary tract organs (excluding the 13 cases with incomplete visualization), focused view CTU intrinsically achieved a sensitivity, specificity, PPV, and NPV of 100.0% (13/13), 92.3% (12/13), 92.9% (13/14), and 100.0% (12/12) for lesions in the urinary tract compared to unmodified unenhanced CT and CTU. The one false positive finding entailed alleged focal wall thickening of the urinary bladder (which was negative on unmodified CTU and cystoscopy). Most lesions concerned urinary stones (Table 3). All 10 incidental findings that were visible on unmodified CTU in the 26 cases with complete visualization were successfully hidden on focused view CTU (Figure 6). Most incidental findings were located in the bowel or pancreas (Table 4).

Interobserver agreement for the detection of urinary tract pathology was moderate (κ = 0.528, *p* = 0.003), with six discrepancies, mostly concerning the kidneys (Table 5). Notably, the second observer missed five cases with a likely cause for hematuria (four were located in the kidney and one in the urinary bladder), which resulted in a sensitivity of 73.7%. Furthermore, the second observer detected one incidental finding (iliac artery aneurysm/widening) that was not detected by the first observer.

## 4. Discussion

The results of this study show the feasibility of a new deep learning-based algorithm that can automatically generate focused view CTU images. This AI system is based on a segmentation of the urinary tract that is enlarged with a 10 mm boundary and the use of an extrapolation method that can bridge interrupted segmentations of the ureters. The added 10 mm segmentation boundary is necessary to be able to evaluate the outer edges of the kidneys, ureters, and urinary bladder, and the extrapolation method is useful to bridge unopacified ureteral segments. Focused view CTU was successful (i.e., complete visualization of the kidneys, ureters, and urinary bladder) in the majority of patients in the initial clinical evaluation analysis, and could detect urinary tract pathology with a diagnostic performance similar to that of unmodified CTU. Only in one case, there was a false positive from the focused view CTU due to presumed focal wall thickening of the urinary bladder, which was negative on prior cystoscopy, and would thus not have any clinical consequences. Meanwhile, all incidental findings that were visible on unmodified CTU were concealed on focused view CTU. However, it should be noted that observer agreement for urinary tract pathology on focused view CTU was moderate. The second observer recorded five false negative cases, resulting in a sensitivity of 73.7% compared to 100% for the first observer. This discrepancy may be attributed to several factors. One plausible explanation is a learning curve effect, as the second observer had no prior experience with segmenting or interpreting focused view CTU scans, unlike the first observer. This lack of familiarity could have limited the second observer’s ability to detect subtle pathologies, even when these were intrinsically visible on the modified CTU images. Furthermore, four of the five missed lesions were located in the kidney and one in the bladder wall, suggesting that the second observer may have been overly focused on evaluating the pyelocalyceal system and ureters, potentially overlooking abnormalities in other parts of the urinary tract. These factors combined may explain the moderate interobserver agreement observed for urinary tract pathology detection using focused view CTU. The findings underscore the importance of adequate training and broader anatomical awareness when interpreting such scans.

Focused view CTU is not perfect yet, with incomplete visualization of the urinary tract in 13/39 (33.3%) subjects. These 13 cases were excluded from the diagnostic performance analysis, which limits the method’s current applicability. Most incomplete segmentations concerned unopacified (distal) ureters, which, despite the use of an added 10 mm boundary and the extrapolation method, were not visualized on focused view CTU. In a future clinical trial setting investigating the relevance of incidental findings on CTU in patients with hematuria, this would necessitate viewing the unmodified CTU, and exclusion of such cases to ensure accurate diagnosis and comprehensive assessment. Future work is desirable to optimize the focused view CTU technique. This may be achieved by improving the segmentation algorithm with a larger training set, or technical innovations. The deep learning code and trained models used in this study are available at an online repository (https://github.com/0xC4/focused-ctu, accessed on 31 July 2025), which can also be used by other researchers to improve the focused view CTU algorithm. Improving the opacification of the ureters at CTU may also be helpful to improve segmentation performance. Although this remains challenging, guidelines and research state that the use of diuretics (furosemide, which was also applied in the present study) before contrast administration [13,14] or increased contrast medium volume [15] may be of value.

Several recent studies applied deep learning algorithms for the segmentation of kidneys or urinary bladders [16,17,18], but none of them have developed a comprehensive approach involving the whole urinary tract that also includes the ureters. In a study by Hadjiiski et al. [19] a computerized system for automated segmentation of ureters on CTU was investigated. However, their method required a manual selection of the proximal ureters on the native CTU scan (which implies that all incidental findings would be visible to the reader), and their segmentation results were not flawless, even though they excluded ureters with poor opacification [20]. Nevertheless, this ureter tracking algorithm as was used by Hadjiiski et al. [19] may potentially be useful for integration in our focused view CTU method. Of interest, a recent study by Roest et al. [20] investigated a similar approach as was used in the present study to obtain a selective visualization of stroke related arteries. However, their focused-view CT angiography method did not include any segmentation boundaries or use extrapolation methods [20], whereas incorporating segmentation boundaries and an extrapolation method could have been beneficial for segmenting small-caliber and/or unopacified arteries, similar to unopacified ureters.

This study had some limitations. First, we did not include patients with prior urinary tract surgeries or interventions such as nephrectomy, cystectomy, kidney transplantation, or ureteral stent placement, which could complicate anatomy. Therefore, the focused view CTU algorithm may demonstrate reduced performance and segmentation accuracy in such complex clinical cases. However, it should be noted that patients with a history of urinary tract surgery or a ureteral stent usually have undergone previous cross-sectional imaging, and the chance of detecting new, clinically significant incidental findings on CTU in such cases can be considered low. In addition, our future target population in which we aim to investigate the relevance of incidental findings on CTU are patients with newly diagnosed hematuria, and not patients who have already undergone a urological procedure, surgery, or prior imaging evaluation. Second, the focused view CTU algorithm was developed based on dual-phase CTU scans, in which the nephrographic and excretory phases were combined in one scan after a split bolus contrast agent injection. However, some institutions use single-bolus techniques, for which the segmentation performance of our model may be different and potentially less accurate or reliable without further adaptation. Third, there may be a learning curve with regard to the interpretation of focused view CTU scans, which was not addressed in the present study and requires further investigation. Fourth, the focused view CTU method was evaluated in a retrospective, experimental setting. Future studies are required to determine its performance in a prospective clinical setting. Fifth, only one observer performed the technical optimization analysis.

Finally, although we argue for a potential future role of focused view CTU, it should be noted that there are several arguments against the use of a focused view approach in clinical practice. Given that CT radiation is applied regardless, discarding most of the acquired data requires ethical justification. Limiting interpretation to a narrow field of view risks missed findings with potential medicolegal consequences. Moreover, comprehensive imaging can be invaluable for future comparisons. Importantly, if additional findings are relevant, their treatment may be more affordable and effective at an early, asymptomatic stage. Alternatively, defining appropriate investigations for incidental findings could help reduce unnecessary follow-up and associated costs. The pros and cons of a focused view approach have been discussed in previous work [21]. Overall, a future prospective randomized trial is needed to evaluate the clinical value of this approach.

## 5. Conclusions

This study demonstrated the feasibility of a novel deep learning-based algorithm for generating focused view CTU, which selectively visualizes the urinary tract while masking extraneous anatomy. The optimized method, incorporating boundary expansion and extrapolation, achieved high visualization rates and comparable diagnostic performance to standard CTU, while concealing incidental findings (excluding one-third of cases in which the visualization of the urinary tract was incomplete). Although incomplete visualization occurred in one-third of cases (which limits the methods current applicability) and interobserver agreement was moderate, focused view CTU shows promise for future clinical trials investigating the relevance of incidental findings.

## Figures and Tables

**Table 1:** Visualization of urinary tract organs with three different methods (method 1: the baseline method, in which the unmodified AI-generated segmentation was used to mask the voxels outside of the segmented regions; method 2: the expanded boundary method in which a binary dilation with a spherical structuring element was used to expand the visualized area with a 10 mm boundary around the segmented; and method 3: the expanded boundary and extrapolation method, which expands on method 2 by bridging interrupted segmentations of the ureters by ensuring that the segmentation contained a connection between both kidneys and the bladder).

Organ	Visualization	Method			Best Method(s)
		1	2	3	
Kidneys (n = 60)	100%	0 (0%)	57 (95%)	57 (95%)	2 and 3 (*p* ≤ 0.001 vs. 1)
	96–99%	59 (98%)	3 (5%)	3 (5%)	
	75–95%	1 (2%)	0 (0%)	0 (0%)	
	<75%	0 (0%)	0 (0%)	0 (0%)	
Proximal ureters (n = 60)	100%	0 (0%)	54 (90%)	57 (95%)	3 (*p* ≤ 0.001 vs. 1) (*p* ≤ 1.000 vs. 2)
	96–99%	54 (90%)	2 (3%)	2 (3%)	
	75–95%	2 (3%)	3 (5%)	0 (0%)	
	<75%	4 (7%)	1 (2%)	1 (2%)	
Mid ureters (n = 60)	100%	0 (0%)	53 (88%)	59 (98%)	3 (*p* ≤ 0.001 vs. 1) (*p* = 1.000 vs. 2)
	96–99%	51 (85%)	3 (5%)	0 (0%)	
	75–95%	5 (8%)	2 (3%)	0 (0%)	
	<75%	4 (7%)	3 (3%)	1 (2%)	
Distal ureters (n = 60)	100%	0 (0%)	41 (68%)	57 (95%)	3 (*p* = <0.001 vs. 1) (*p* = 0.249 vs. 2)
	96–99%	40 (67%)	10 (17%)	1 (2%)	
	75–95%	11 (18%)	8 (13%)	2 (3%)	
	<75%	9 (15%)	1 (2%)	0 (0%)	
Bladder (n = 30)	100%	0 (0%)	24 (80%)	26 (87%)	3 (*p* ≤ 0.001 vs. 1) (*p* = 1.000 vs. 2)
	96–99%	29 (97%)	5 (17%)	3 (10%)	
	75–95%	1 (3%)	1 (3%)	1 (3%)	
	<75%	0 (0%)	0 (0%)	0 (0%)	

**Table 2:** Visualization of urinary tract organs in the initial clinical evaluation.

Organ	Completely Visualized	Substantial Part Not Visualized	Miniscule Part Not Visualized
Kidneys (n = 78)	76 (97.4%)	2 (2.6%)	0 (0.0%)
Ureters (n = 78)	63 (80.8%)	12 (15.4%)	3 (3.8%)
Bladder (n = 39)	37 (94.8%)	1 (2.6%)	1 (2.6%)

**Table 3:** Potentially hematuria-causing lesions on scans of subjects used for initial clinical evaluation (n = 13).

Lesion	No.
Urolithiasis	6
Tumor urinary bladder	5
Nephritis	1
Tumor kidney	1

**Table 4:** Extra-urinary tract incidental findings on scans of subjects used for initial clinical evaluation (n = 10), which were not visible on focused CTU view.

Location of Incidental Finding	No.
Bowel	2
Pancreas	2
Iliac arteries	1
Liver	1
Lungs	1
Lymph node	1
Retroperitoneal space	1
Other (signs of portal hypertension)	1

**Table 5:** Discrepancies between observers for urinary tract pathology.

Location	Description	Outcome
Kidney	4 mm stone	False negative second observer
Kidney	3 mm stone	False negative second observer
Kidney	Focal hypoattenuation, likely focal nephritis	False negative second observer
Kidney	Tumor, likely renal cell carcinoma	False negative second observer
Urinary bladder	Diffuse wall thickening due to tumor	False negative second observer
Urinary bladder	Focal wall thickening	False positive first observer

## Data Availability

The data are available upon reasonable request from the authors.

## Supplementary Materials

The following supporting information can be downloaded at: https://www.mdpi.com/article/10.3390/diseases13080242/s1, Video S1: Axial enhanced and unenhanced CT (top left, top right) and focused view enhanced and unenhanced CT (bottom left, bottom right) slices for a patient included in the initial clinical evaluation cohort. Video S2: Coronal enhanced and unenhanced CT (top left, top right) and focused view enhanced and unenhanced CT (bottom left, bottom right) slices for a patient included in the initial clinical evaluation cohort.

## References

[1] Mariani A.J., Mariani M.C., Macchioni C., Stams U.K., Hariharan A., Moriera A. (1989). The significance of adult hematuria: 1,000 hematuria evaluations including a risk-benefit and cost-effectiveness analysis. J. Urol..

[2] Davis R., Jones J.S., Barocas D.A., Castle E.P., Lang E.K., Leveillee R.J., Messing E.M., Miller S.D., Peterson A.C., Turk T.M.T. (2012). Diagnosis, evaluation and follow-up of asymptomatic microhematuria (AMH) in adults: AUA guideline. J. Urol..

[3] Rai B.P., Luis Dominguez Escrig J., Vale L., Kuusk T., Capoun O., Soukup V., Bruins H.M., Yuan Y., Violette P.D., Santesso N. (2022). Systematic review of the incidence of and risk factors for urothelial cancers and renal cell carcinoma among patients with haematuria. Eur. Urol..

[4] Bromage S.J., Liew M.P., Moore K.C., Raju B., Shackley D.C. (2012). The economic implications of unsuspected findings from CT urography performed for haematuria. Br. J. Radiol..

[5] Morgan A.E., Berland L.L., Ananyev S.S., Lockhart M.E., Kolettis P.N. (2015). Extraurinary incidental findings on CT for hematuria: The radiologist’s role and downstream cost analysis. AJR Am. J. Roentgenol..

[6] Kwee R.M., Kwee T.C. (2019). Whole-body MRI for preventive health screening: A systematic review of the literature. J. Magn. Reson. Imaging.

[7] Lai W.S., Ellenburg J., Lockhart M.E., Kolettis P.N. (2016). Assessing the costs of extraurinary findings of computed tomography urogram in the evaluation of asymptomatic microscopic hematuria. Urology.

[8] Davenport M.S. (2023). Incidental findings and low-value care. AJR Am. J. Roentgenol..

[9] Scott I.A., Slavotinek J., Glasziou P.P. (2024). First do no harm in responding to incidental imaging findings. Med. J. Aust..

[10] Ronneberger O., Fischer P., Brox T., Navab N., Hornegger J., Wells W., Frangi A. (2015). U-Net: Convolutional networks for biomedical image segmentation. Medical Image Computing and Computer-Assisted Intervention—MICCAI 2015.

[11] Isensee F., Jaeger P.F., Kohl S.A.A., Petersen J., Maier-Hein K.H. (2021). nnU-Net: A self-configuring method for deep learning-based biomedical image segmentation. Nat. Methods.

[12] Yushkevich P.A., Piven J., Hazlett H.C., Smith R.G., Ho S., Gee J.C., Gerig G. (2006). User-guided 3D active contour segmentation of anatomical structures: Significantly improved efficiency and reliability. Neuroimage.

[13] Renard-Penna R., Rocher L., Roy C., André M., Bellin M.-F., Boulay I., Eiss D., Girouin N., Grenier N., Hélénon O. (2020). Imaging protocols for CT urography: Results of a consensus conference from the French Society of Genitourinary Imaging. Eur. Radiol..

[14] Ascenti G., Cicero G., Cardone G., Bertelli E., Papa M., Ciccone V., Manetta R., Gentili F., Francioso A.P., Mazzei M.A. (2023). Cornerstones of CT urography: A shared document by the Italian board of urogenital radiology. Radiol. Med..

[15] Szolar D.H., Tillich M., Preidler K.W. (2010). Multi-detector CT urography: Effect of oral hydration and contrast medium volume on renal parenchymal enhancement and urinary tract opacification—a quantitative and qualitative analysis. Eur. Radiol..

[16] Lin Z., Cui Y., Liu J., Sun Z., Ma S., Zhang X., Wang X. (2021). Automated segmentation of kidney and renal mass and automated detection of renal mass in CT urography using 3D U-Net-based deep convolutional neural network. Eur. Radiol..

[17] Cha K.H., Hadjiiski L., Samala R.K., Chan H.P., Caoili E.M., Cohan R.H. (2016). Urinary bladder segmentation in CT urography using deep-learning convolutional neural network and level sets. Med. Phys..

[18] Ma X., Hadjiiski L.M., Wei J., Chan H.-P., Cha K.H., Cohan R.H., Caoili E.M., Samala R., Zhou C., Lu Y. (2019). U-Net based deep learning bladder segmentation in CT urography. Med. Phys..

[19] Hadjiiski L., Zick D., Chan H.P., Cohan R.H., Caoili E.M., Cha K., Zhou C., Wei J. (2014). Ureter tracking and segmentation in CT urography (CTU) using COMPASS. Med. Phys..

[20] Roest C., Kloet R.W., Lamers M.J., Yakar D., Kwee T.C. (2023). Focused view CT angiography for selective visualization of stroke related arteries: Technical feasibility. Eur. Radiol..

[21] Kwee T.C., Yakar D., Sluijter T.E., Pennings J.P., Roest C. (2023). Can we revolutionize diagnostic imaging by keeping Pandora’s box closed?. Br. J. Radiol..

